# Caution in inferring viral strategies from abundance correlations in marine metagenomes

**DOI:** 10.1038/s41467-018-07950-z

**Published:** 2019-01-30

**Authors:** Hend Alrasheed, Rong Jin, Joshua S. Weitz

**Affiliations:** 1 School of Biological Sciences, Georgia Institute of Technology, Atlanta, GA 30332 USA; 2 School of Physics, Georgia Institute of Technology, Atlanta, GA 30332 USA

**Arising from** F.H. Coutinho et al. *Nature Communications* 10.1038/ncomms15955 (2017).

Coutinho et al.^1^ reported metagenomics-derived evidence in support of the ‘Piggyback-the-Winner’ (PtW) hypothesis that lysogeny prevalence increases at high microbial abundances. Coutinho et al.^1^ did not directly estimate lysogenic prevalence, but instead, found that the ratio of virus-to-microbial host abundances decreased as microbial cell abundances increased. This pattern represents potential (albeit indirect) evidence in support of PtW. Here, we show that the bulk of these reported abundance relationships are likely spurious. Instead, we find absence of evidence for positive, sublinear correlations between virus and microbial abundances as estimated in dozens of putative virus-microbe pairs identified by Coutinho et al.^1^ The absence of correlations between virus and microbial abundances is a counter-indicator for PtW. Altogether, our re-analysis suggests the need for caution in using correlation-based inference to identify viral strategies from metagenomics-derived abundance relationships.

To begin, consider the work of Coutinho et al.^1^, who developed a metagenomics-based approach to characterize the diversity, ecology, host-associations, and strategies of marine phage. In doing so, they introduced a “new method for host prediction based on co-occurrence associations”, in which “virus–virus abundance associations were used for host affiliation”^1^. As a result, Coutinho et al.^1^ claimed that observed abundance relationships amongst phage and bacterial hosts in a range of marine habitats are consistent with the recently introduced mechanism of PtW^2^.

The hypothesis underlying PtW is that viruses have increased lysogenic prevalence (and decreased lytic activity) with increasing microbial abundances. This hypothesis is meant to provide a mechanistic basis for empirical findings that total virus abundances increase with total microbial abundances even as the number of viruses per microbe decreases as microbial abundances increase. This pattern is found across marine, freshwater and other environmental systems (see Knowles et al.^2^, Wigington et al.^3^, and Parikka et al.^4^), with similar patterns found in predator–prey relationships^5^.

Sublinear (or less than proportional^6^) increases in virus abundances with microbial abundances may arise from multiple governing mechanisms. These mechanisms include PtW, whose underlying mathematical model predicts that viral abundances increase with increasing microbial abundances, albeit sublinearly (see Fig. 1b of ref. ^2^). Addditional mechanisms that could explain sublinear increases include variation in life history traits in antagonistic virus–microbe dynamics^7^ or trade-offs in Kill-the-Winner models^8^. As a consequence, the value of these patterns as exclusive indicators of any particular mechanism is disputed (see exchange of Weitz et al.^7^ and Knowles and Rohwer^9^, as well as the follow-up work of Knowles et al.^10^). Nonetheless, the possibility of using metagenomics-based methods to infer virus–host pairs and their abundance relationships could provide insights into viral strategies and their consequences in marine systems.

**Fig. 1:**
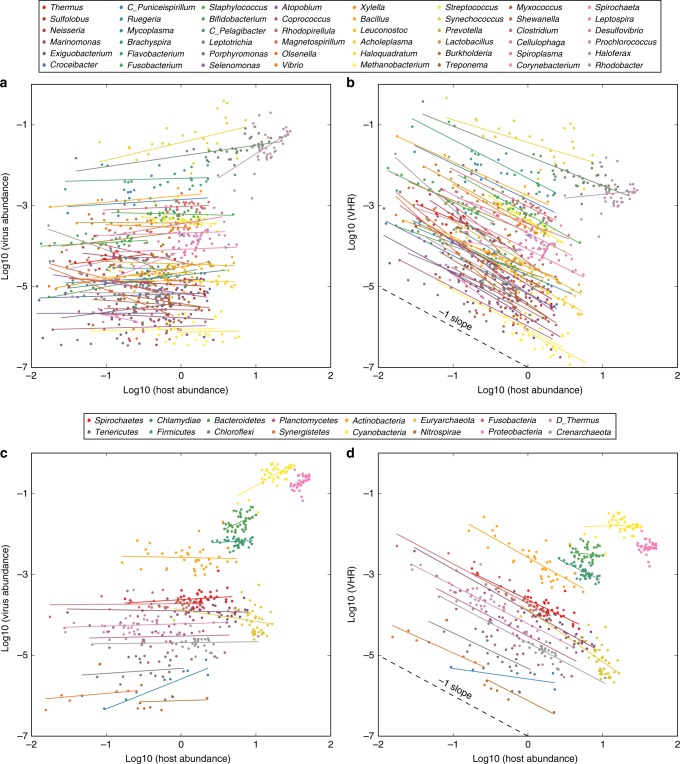
Relationships between virus abundances, virus–host ratios (VHRs), and host abundances. **a** Genus-level relationships, virus vs. host abundances. **b** Genus-level relationships, VHRs vs. host abundances. **c** Phylum-level relationships, virus vs. host abundances. **d** Phylum-level relationships, VHRs vs. host abundances. Panels **b** and **d** are replotted from ref. ^1^. The absence of a relationship between original, untransformed virus and host abundances (see panels **a** and **c**) should appear as a −1 slope when comparing VHR to host abundances (see panels **b** and **d**). A −1 slope is shown as a guide to readers in panels **b** and **d**

Here, we focus on the empirical findings of Coutinho et al.^1^ and ask: do the abundance relationships exhibit robust evidence for sublinear increases in virus abundances with microbial abundances? Coutinho et al.^1^ used multiple approaches, including virus–virus abundance associations, to link viruses and their putative hosts. We use the term “abundances” as a proxy for the metagenomics-inferred relative densities of viruses and host types reported by Coutinho et al.^1^, consistent with their implementation. Once they estimated abundances, Coutinho et al.^1^ quantified the relationship between the ratio of virus-to-host abundances vs. host abundances given putative pairs at both the genus and phylum levels. For example, let *y* be the log-transformed virus abundance and *x* be the log-transformed host abundance of an identified pair. If *y* increased sublinearly with *x*, then one would expect that *y* ~ *x*^*α*^ where 0 < *α* < 1. The inequality *α* > 0 implies that virus abundances increase with microbial abundances and the inequality *α* < 1 implies that the increase is sublinear.

It is also possible to evaluate ratio-based fits, i.e., quantifying the relationship between *y*/*x* and *x*. In that case, we expect *y*/*x* ~ *x*^*β*^ where *β* = *α* − 1. Hence, sublinear power-law relationships between *y* and *x* should lead to power-law relationships between virus-microbe ratios and microbial abundances with negative slopes between −1 < *β* < 0. Coutinho et al.^1^ examined relationships between *y*/*x* vs. *x*, rather than directly examining *y* vs. *x*. If *y* is unrelated to *x* then one would expect best-fit curves between *y*/*x* vs. *x* to be statistically equivalent to fitting 1/*x* vs. *x*, thereby yielding a slope of *β* = −1 on a log–log plot. This relationship is an example of spurious self-correlation, in which inferences are derived based on correlating *x* against itself, in the absence of supporting evidence that *y* is correlated with *x* (see refs. ^11,12^). Upon initial inspection, many reported slopes in Coutinho et al.^1^ appear to be close to −1 (see re-plot of data in Fig. 1b, d).

This observation forms the basis for the present analysis. If slopes of *y*/*x* vs. *x* are in fact indistinguishable from −1, then it would seem to indicate that there is not evidence that virus abundances, *y*, increase with microbial abundances, *x*. Moreover, we should not conclude that the power-law exponent *α* is greater than zero. Initial inspection suggests that there is a systematic absence of evidence for a relationship between *y* and *x* (see Fig. 1a, c). A lack of positive correlation would be surprising and not supportive of PtW (or the empirical literature) irrespective of whether or not the virus-to-microbe ratio decreased as microbes increased. In essence, by focusing on the inequality *β* < 0, it appears Coutinho et al.^1^ did not fully consider evidence that *α* > 0 (in a statistically significant sense).

To investigate this further, we evaluated the statistical relationship between *y* and *x* using a permutation test. The permutation test takes each host–virus pair and then permutes the virus abundances randomly, while maintaining the same host abundances. After each permutation, we recalculated the correlation between virus and host abundances across metagenomes. Such permutations should, in principle, eliminate latent correlations between the two variables. Yet, by chance, we might find some correlations even after permutation. Hence, this test enables a quantitative answer to the question: how often should there be slopes with at least as large a magnitude as observed in the data in the event that there were no underlying relationship between virus and host abundances? We can apply similar permutation methods for nonparametric slopes. In practice, we implemented a two-tailed randomized permutation test to generate a sampling distribution of expected slopes with mean 0.

We find that only 6 genus datasets (out of 48) and 4 phylum datasets (out of 16) have significant correlations at the *p* = 0.05 level (see Fig. 2). A strict Bonferroni correction suggests a criterion for significance of *p* = 0.00078 given the 64 (48 + 16) total comparisons. Hence, the threshold of *p* = 0.05 is permissive. Whether using *p* = 0.05 or *p* = 0.01 for product–moment or rank-based correlations, our results suggest that nearly all of the relationships reported in Coutinho et al.^1^, do not show evidence of a relationship between virus and host abundance and therefore do not provide even indirect support for PtW (see Supplementary Tables 1–4 for confidence intervals for all relationships examined). Critically, our findings are consistent with calculations of the significance of virus–host correlations reported in Supplementary Tables 3 and 4 of Coutinho et al.^1^, who nonetheless claimed that their “findings corroborate the recently proposed Piggyback-the-Winner theory.”

**Fig. 2:**
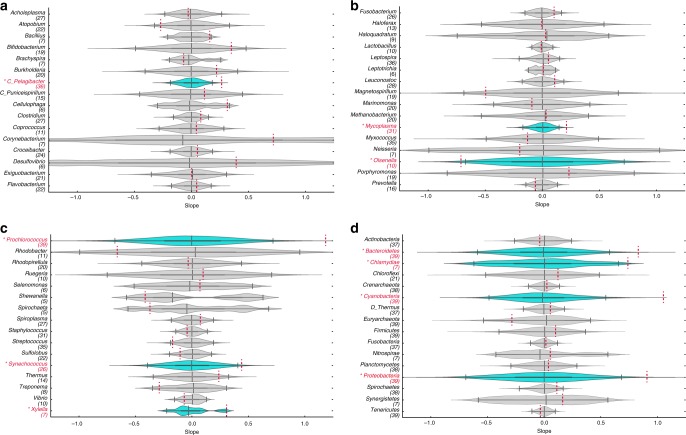
Violin plots of the 95% randomized confidence intervals (CIs) of the slope for relationships between virus and host abundances. **a**–**c** CIs in genus datasets. **d** CIs in phylym datasets. In each panel, red dashed lines indicate the slope of the original sample. Dark vertical lines indicate the 2.5, 50, and 97.5 percentiles. Datasets with slopes with *p* ≥ 0.05 are shown in gray violin plots and datasets with slopes with *p* < 0.05 are shown in blue violin plots. The number of data points for each dataset is indicated in the parenthesis below the genus/phylum name

Given this reanalysis, we conclude that the abundance relationships derived from marine metagenomics datasets in Coutinho et al.^1^ do not have robust evidence in support of PtW. To the contrary, nearly the entire dataset of putative relationships between viruses and hosts do not have evidence that virus abundances are related to host abundances. It is this lack of a relationship (i.e., *α* ≈ 0) that explains why the observation that the ratio of viruses to hosts decreases with increasing host abundances (i.e., *β* ≈ −1) was incorrectly interpreted in Coutinho et al.^1^. Such a decrease can occur in the absence of a relationship between virus abundances and host abundances. This has been termed spurious self-correlation^11,12^.

Our reanalysis raises a number of questions. The absence of significant abundance relationships between viruses and hosts may be a consequence of analyzing correlations based on relative abundances, which can lead to spurious findings^13^. The absence of a relationship may also indicate that the method for host prediction developed by Coutinho et al.^1^ fails to identify genuine interactions. In that event, the absence of a correlation may reflect the absence of an underlying interaction between putative virus–host pairs. However, correlation does not imply causation, just as the absence of correlation does not imply the absence of causation. Multiple studies have reported that correlation in microbe–microbe dynamics and virus–microbe dynamics may be a poor indicator of interaction^14–16^.

We recognize that correlation-based inference of virus–host interactions was one of multiple methods used by Coutinho et al.^1^ to identify virus–host pairs. If the methods of Coutinho et al.^1^ accurately identify virus–host pairs, then our re-examination suggests that abundance relationships between viruses and microbes may differ starkly when considered amongst lineages^1^ vs. when given total abundance data^2–4^. If robust, the absence of a significant relationship between virus and microbial abundances given lineage-specific interactions may result from other eco-evolutionary drivers, e.g., “cryptic dynamics” in which rapid evolution masks latent abundance relationships^17^.

In summary, we cannot say definitively whether the identified virus–microbes associations in Coutinho et al.^1^ are functionally relevant. However, we can conclude that the abundance relationships inferred from metagenomes do not provide robust, indirect support for PtW (or other mechanistic hypotheses) that predict sublinear increases in viral abundances with microbial abundances. Moving forward, we hope that the combined use of new in situ technologies and principled, in silico analyses can help advance the identification of likely virus–host interactions, viral strategies, and population-level consequences of viral infection.

## Methods

### Datasets

The datasets of Coutinho et al.^1^ were partitioned based on whether interactions were identified at the genus or phylum scale. In the original dataset, there were 17 and 93 virus–host datasets at the phylum scale and genus scale respectively. Each dataset has 39 distinct sampling sites. However, those sites with zero virus abundance or zero host abundance were excluded. In addition, we did not include any datasets for which there were not at least five sample sites with both non-zero levels of microbes and viruses. As a result, the datasets analyzed included 16 of 17 original virus–host pairs at the phylum level and 48 of 93 original virus–host pairs at the genus level.

### Statistical analysis

We implemented permutation tests to assess measured correlations between virus and host abundances. For each host dataset we generated 10^4^ randomized samples of size *n*_*i*_ (the number of sample sites for virus–host pair *i*). In each sample, we permuted the virus abundances without replacement while holding the host abundances fixed. Distributions of measured correlations were compared to the original samples using product–moment correlation (main text and Supplementary Information) and Spearman rank correlation (see Supplementary Information).

## Supplementary Information

Supplementary Information

## Data Availability

The original data is from Coutinho et al.^1^. All statistical analysis code and results are available via an open access link at 10.5281/zenodo.1478122.
