# Author Correction: Caution in inferring viral strategies from abundance correlations in marine metagenomes

**DOI:** 10.1038/s41467-019-08955-y

**Published:** 2019-02-19

**Authors:** Hend Alrasheed, Rong Jin, Joshua S. Weitz

**Affiliations:** 1 School of Biological Sciences, Georgia Institute of Technology, Atlanta, GA 30332 USA; 2 School of Physics, Georgia Institute of Technology, Atlanta, GA 30332 USA

Correction to: *Nature Communications*; 10.1038/s41467-018-07950-z, Published online 30 January 2019

The original version of the Article contained a spelling error in the word ‘piggyback’. This error has been corrected in both the PDF and HTML versions of the Article.

