# Supplementary material for: Caution in inferring viral strategies from abundance correlations in marine metagenomes
Source: Nat Commun. 2019 Jan 30;10:501. doi: 10.1038/s41467-018-07950-z (PMC6353933; doi:10.1038/s41467-018-07950-z)
Supplement: Supplementary file 1 — Supplementary Information [file 41467_2018_7950_MOESM1_ESM.pdf]

Caution in inferring viral strategies from abundance correlations in marine metagenomes

Hend Alrasheed[1], Rong Jin[1], and Joshua S. Weitz[1,2]
[1]School of Biological Sciences and [2]School of Physics
Georgia Institute of Technology, Atlanta, GA, 30332, USA

Supplementary Materials

- Table S1: Slope correlation distributions for the genus datasets.
- Table S2: Slope correlation distributions for the phylum datasets.
- Table S3: Spearman rank correlation distributions for the genus datasets.
- Table S4: Spearman rank correlation distributions for the phylum datasets.

Table S1: Randomized confidence intervals of slope for the genus datasets. * indicates datasets that show significant correlation at the $p = 0.05$ level. ** indicates datasets that show significant correlation at the $p = 0.01$ level.

Host	# of data points	Original sample		Randomized sample distribution				
		Slope	p-value	Median slope	95% CI		99% CI	
Acholeplasma	27	-0.0276	0.8331	-0.0057	-0.2347	0.2700	-0.2940	0.3624
Atopobium	22	-0.2696	0.1169	-0.0011	-0.3258	0.3380	-0.4056	0.4406
Bacillus	7	0.1604	0.1269	0.0058	-0.2048	0.1768	-0.2315	0.2104
Bifidobacterium	19	0.3506	0.1651	0.0046	-0.4817	0.4805	-0.6263	0.5980
Brachyspira	7	-0.0679	0.6290	-0.0218	-0.1908	0.2590	-0.2145	0.2737
Burkholderia	20	0.2206	0.3088	-0.0051	-0.3986	0.4131	-0.5139	0.5277
C_Pelagibacter**	36	0.2651	0.0030	0.0009	-0.1852	0.1826	-0.2293	0.2317
C_Puniceispirillum	15	0.1150	0.6456	-0.0012	-0.4591	0.4610	-0.5709	0.5745
Cellulophaga	6	0.3157	0.0510	-0.0097	-0.2934	0.3343	-0.3210	0.3496
Clostridium	27	0.0819	0.3130	0.0008	-0.1625	0.1471	-0.2083	0.1871
Coprococcus	11	0.0443	0.7822	0.0016	-0.2821	0.2879	-0.3470	0.3483
Corynebacterium	7	0.7186	0.5267	-0.0717	-1.8077	1.7605	-1.9219	1.8860
Croceibacter	24	0.0537	0.5899	-0.0008	-0.1858	0.1900	-0.2334	0.2420
Desulfovibrio	6	0.3933	0.6670	-0.0171	-1.3452	1.5003	-1.4183	1.5905
Exiguobacterium	21	0.0033	0.9851	0.0129	-0.3516	0.3118	-0.4626	0.3933
Flavobacterium	22	0.0463	0.6201	0.0010	-0.1786	0.1720	-0.2271	0.2170
Fusobacterium	26	0.1035	0.2143	-0.0023	-0.1490	0.1656	-0.1835	0.2174
Haloferax	13	-0.0046	0.9879	0.0013	-0.5392	0.5507	-0.6696	0.6631
Haloquadratum	9	0.0292	0.9403	0.0435	-0.7410	0.5710	-0.8207	0.6908
Lactobacillus	10	-0.0085	0.8732	-0.0012	-0.0896	0.0944	-0.1095	0.1129
Leptospira	36	0.0543	0.5468	0.0068	-0.2070	0.1553	-0.2811	0.1970
Leptotrichia	6	0.0095	0.9062	0.0074	-0.1186	0.1155	-0.1288	0.1288
Leuconostoc	28	0.1078	0.2479	-0.0045	-0.1800	0.1821	-0.2285	0.2320
Magnetospirillum	19	-0.4958	0.1582	0.0081	-0.6846	0.6657	-0.8587	0.8290
Marinomonas	20	-0.0909	0.6871	0.0002	-0.4209	0.4145	-0.5345	0.5125
Methanobacterium	20	0.0339	0.8910	0.0254	-0.5298	0.4087	-0.6452	0.5159
Mycoplasma**	31	0.2115	0.0061	0.0021	-0.1728	0.1479	-0.2173	0.1870
Myxococcus	35	-0.1295	0.6175	0.0022	-0.5001	0.4822	-0.6383	0.6195
Neisseria	7	-0.1975	0.7513	0.0047	-0.9925	1.0273	-1.1130	1.1478
Olsenella*	10	-0.7128	0.0434	-0.0136	-0.6664	0.6963	-0.8307	0.8406
Porphyromonas	19	0.2335	0.5947	0.0105	-0.8129	0.8137	-1.0427	1.0022
Prevotella	16	-0.0605	0.4226	0.0005	-0.1434	0.1376	-0.1800	0.1743
Prochlorococcus**	39	1.1848	0.0004	-0.0111	-0.6898	0.7088	-0.8749	0.9613
Rhodobacter	11	-0.6610	0.2201	0.0174	-1.0059	0.9626	-1.2080	1.1494
Rhodopirellula	20	-0.0324	0.8896	-0.0001	-0.4314	0.4406	-0.5527	0.5526
Ruegeria	10	0.0993	0.8130	0.0132	-0.7289	0.7006	-0.8829	0.8264
Selenomonas	6	0.0714	0.8341	-0.0105	-0.5078	0.5365	-0.5815	0.5961
Shewanella	5	-0.4136	0.3683	-0.1669	-0.5762	0.6300	-0.5830	0.6427
Spirochaeta	5	-0.3717	0.3494	-0.0636	-0.5656	0.6806	-0.5840	0.6857
Spiroplasma	27	0.0754	0.4434	0.0010	-0.1820	0.1922	-0.2282	0.2422
Staphylococcus	31	-0.0421	0.5624	0.0012	-0.1383	0.1397	-0.1773	0.1762
Streptococcus	35	-0.1717	0.0833	-0.0013	-0.1950	0.1902	-0.2496	0.2395
Sulfolobus	22	-0.1043	0.2847	0.0045	-0.2001	0.1741	-0.2498	0.2184
Synechococcus*	26	0.4406	0.0311	0.0101	-0.3961	0.4050	-0.4987	0.5218
Thermus	14	0.2381	0.1727	0.0002	-0.3321	0.3308	-0.4158	0.4118
Treponema	8	-0.2886	0.1016	0.0156	-0.3510	0.3098	-0.4181	0.3624
Vibrio	10	-0.0673	0.4078	0.0080	-0.1620	0.1274	-0.1958	0.1470
Xylella*	7	0.3073	0.0055	-0.0294	-0.2245	0.3035	-0.2480	0.3182

Table S2: Randomized confidence intervals of slope for the phylum datasets. * indicates datasets that show significant correlation at the $p = 0.05$ level. ** indicates datasets that show significant correlation at the $p = 0.01$ level.

Host	# of data points	Original sample		Randomized sample distribution				
		Slope	p-value	Median slope	95% CI		99% CI	
Actinobacteria	37	-0.0387	0.7707	0.0023	-0.2586	0.2442	-0.3272	0.3100
Bacteroidetes**	39	0.8321	0.0041	-0.0055	-0.5826	0.5808	-0.7344	0.7658
Chlamydiae**	7	0.7399	0.0075	0.0006	-0.6170	0.6395	-0.7187	0.7155
Chloroflexi	21	0.1228	0.6487	0.0089	-0.5191	0.4910	-0.6466	0.6288
Crenarchaeota	38	0.0248	0.7256	0.0011	-0.1390	0.1337	-0.1856	0.1688
Cyanobacteria**	39	1.0542	0	-0.0134	-0.4882	0.5638	-0.6186	0.7538
D_Thermus	37	0.0556	0.5552	-0.0014	-0.1843	0.1818	-0.2347	0.2378
Euryarchaeota	39	-0.2846	0.2426	0.0174	-0.5383	0.4083	-0.6841	0.5018
Firmicutes	39	0.1009	0.6144	0.0106	-0.4091	0.3720	-0.5437	0.4716
Fusobacteria	37	0.0158	0.7962	-0.0002	-0.1192	0.1190	-0.1583	0.1486
Nitrospirae	7	0.0545	0.8638	-0.0368	-0.4254	0.5590	-0.4767	0.6372
Planctomycetes	38	0.0386	0.8012	-0.0015	-0.2968	0.2901	-0.3839	0.3797
Proteobacteria**	39	0.9080	0.0092	-0.0032	-0.6980	0.6778	-0.8920	0.8887
Spirochaetes	38	0.1123	0.2590	0.0061	-0.2213	0.1780	-0.2987	0.2288
Synergistetes	7	0.1633	0.6588	0.0213	-0.5803	0.5610	-0.6716	0.6496
Tenericutes	39	-0.0327	0.5388	0.0010	-0.1071	0.0999	-0.1365	0.1226

Table S3: Randomized confidence intervals of Spearman rank for the genus datasets. * indicates datasets that show significant correlation at the $p = 0.05$ level. ** indicates datasets that show significant correlation at the $p = 0.01$ level.

Host	# of data points	Original sample Spearman rank	Randomized sample distribution Median Spearman rank	95% CI		99% CI	
Acholeplasma	27	-0.0956	-0.0006	-0.3777	0.3802	-0.4879	0.4840
Atopobium	22	-0.3604	0.0023	-0.4326	0.4191	-0.5439	0.5445
Bacillus	7	0.6429	0	-0.7500	0.7509	-0.8929	0.8929
Bifidobacterium	19	0.2000	0.0053	-0.4596	0.4509	-0.5842	0.5825
Brachyspira	7	0	0	-0.7857	0.7857	-0.8929	0.8929
Burkholderia	20	0.0707	-0.0045	-0.4361	0.4391	-0.5639	0.5669
C_Pelagibacter**	36	0.7207	0	-0.3326	0.3318	-0.4242	0.4237
C_Puniceispirillum	15	0.0536	0	-0.5214	0.5250	-0.6643	0.6500
Cellulophaga	6	0.6000	0.0286	-0.8286	0.8286	-0.9429	0.9429
Clostridium	27	0.1606	0.0018	-0.3791	0.3700	-0.4799	0.4695
Coprococcus	11	0.1455	0	-0.6000	0.6091	-0.7545	0.7455
Corynebacterium	7	-0.0714	0	-0.7500	0.7500	-0.8929	0.8929
Croceibacter	24	0.2296	0.0009	-0.3974	0.4070	-0.4965	0.5191
Desulfovibrio	6	-0.2000	-0.0286	-0.8286	0.8286	-0.9429	0.9429
Exiguobacterium	21	-0.0208	0.0029	-0.4333	0.4365	-0.5671	0.5495
Flavobacterium	22	0.1282	0.0006	-0.4252	0.4286	-0.5449	0.5505
Fusobacterium	26	0.3415	0.0024	-0.3962	0.3853	-0.5050	0.4920
Haloferax	13	-0.0934	0.0055	-0.5604	0.5604	-0.7033	0.6758
Haloquadratum	9	-0.3167	0	-0.6667	0.6667	-0.8333	0.8000
Lactobacillus	10	-0.0303	0.0061	-0.6364	0.6364	-0.7939	0.7818
Leptospira	36	-0.0013	-0.0005	-0.3287	0.3326	-0.4204	0.4203
Leptotrichia	6	-0.0286	-0.0286	-0.8286	0.8286	-0.9429	0.9429
Leuconostoc	28	0.3082	-0.0060	-0.3771	0.3766	-0.4926	0.4954
Magnetospirillum	19	-0.2632	0.0053	-0.4579	0.4632	-0.5860	0.5755
Marinomonas	20	-0.1053	-0.0015	-0.4301	0.4467	-0.5579	0.5579
Methanobacterium	20	-0.0586	-0.0015	-0.4496	0.4466	-0.5669	0.5805
Mycoplasma*	31	0.3575	-0.0025	-0.3615	0.3549	-0.4621	0.4478
Myxococcus	35	-0.0050	0.0031	-0.3353	0.3297	-0.4317	0.4266
Neisseria	7	-0.2143	0	-0.7500	0.7500	-0.8929	0.8929
Olsenella**	10	-0.8061	-0.0061	-0.6364	0.6364	-0.7819	0.7697
Porphyromonas	19	0.2140	0.0035	-0.4561	0.4614	-0.5895	0.5930
Prevotella	16	-0.1824	-0.0029	-0.4882	0.5059	-0.6265	0.6500
Prochlorococcus**	39	0.4063	-0.0024	-0.3190	0.3148	-0.4091	0.4006
Rhodobacter	11	-0.5000	0	-0.6000	0.6091	-0.7364	0.7364
Rhodopirellula	20	-0.0120	0.0015	-0.4421	0.4466	-0.5684	0.5669
Ruegeria	10	0.1030	0.0061	-0.6364	0.6242	-0.7455	0.7697
Selenomonas	6	-0.1429	0.0286	-0.8286	0.8286	-0.9429	0.9429
Shewanella	5	-0.1000	0	-0.9000	0.9000	-1.0000	1.0000
Spirochaeta	5	-0.5643	-0.0513	-0.8721	0.8721	-0.9747	0.9747
Spiroplasma	27	0.2393	0.0055	-0.3816	0.3846	-0.4890	0.4927
Staphylococcus	31	-0.0931	0.0032	-0.3536	0.3593	-0.4677	0.4532
Streptococcus	35	-0.3129	-0.0006	-0.3359	0.3364	-0.4429	0.4384
Sulfolobus	22	-0.4155	-0.0006	-0.4200	0.4200	-0.5466	0.5308
Synechococcus*	26	0.4865	0.0085	-0.3826	0.3929	-0.4872	0.5036
Thermus**	14	0.6967	-0.0066	-0.5430	0.5341	-0.6747	0.6747
Treponema	8	-0.6667	0	-0.7143	0.7143	-0.8571	0.8571
Vibrio	10	-0.3697	0.0061	-0.6245	0.6364	-0.7697	0.7697
Xylella	7	0.6429	0	-0.7500	0.7500	-0.8929	0.8929

Table S4: Randomized confidence intervals of Spearman rank for the phylum datasets. * indicates datasets that show significant correlation at the $p = 0.05$ level. ** indicates datasets that show significant correlation at the $p = 0.01$ level.

Host	# of data points	Original sample Spearman rank	Randomized sample distribution				
			Median Spearman rank	95% CI		99% CI	
Actinobacteria	37	-0.1083	0.0031	-0.3293	0.3208	-0.4218	0.4166
Bacteroidetes**	39	0.4879	-0.0019	-0.3158	0.3156	-0.4022	0.4117
Chlamydiae*	7	0.8571	0	-0.7500	0.7500	-0.8929	0.8571
Chloroflexi	21	0.1403	0	-0.4390	0.4338	-0.5533	0.5546
Crenarchaeota	38	0.0780	0.0005	-0.3177	0.3252	-0.4125	0.4187
Cyanobacteria**	39	0.4287	0.0026	-0.3117	0.3148	-0.4081	0.4273
D_Thermus	37	0.0512	-0.0019	-0.3284	0.3184	-0.4163	0.4111
Euryarchaeota*	39	-0.3381	0.0004	-0.3166	0.3109	-0.4107	0.4073
Firmicutes	39	0.0496	0.0011	-0.3190	0.3152	-0.4235	0.4128
Fusobacteria	37	-0.0441	-0.0017	-0.3205	0.3269	-0.4258	0.4227
Nitrospirae	7	0	0	-0.7500	0.7500	-0.8929	0.8929
Planctomycetes	38	0.0299	-0.0023	-0.3269	0.3220	-0.4251	0.4199
Proteobacteria**	39	0.5700	-0.0016	-0.3164	0.3097	-0.4069	0.3945
Spirochaetes	38	0.2520	-0.0012	-0.3155	0.3207	-0.4041	0.4264
Synergistetes	7	0.3214	0	-0.7857	0.7500	-0.8929	0.8929
Tenericutes	39	-0.1405	-0.0010	-0.3130	0.3166	-0.4105	0.4024
